# Study on the Compressive Mechanical Behavior of Multi-Segment Spliced Beams for Hybrid Prefabricated Reinforced Concrete–Steel Structure Foundation Pit Bracing System

**DOI:** 10.3390/ma19142997

**Published:** 2026-07-11

**Authors:** Kaijun Xu, Jie Chen, Houmin Li, Jianjun Ye

**Affiliations:** 1 College for Elite Engineers, China University of Geosciences, Wuhan 430074, China; 2 School of Civil Engineering, Architecture and the Environment, Hubei University of Technology, Wuhan 430068, China; 3 Wuhan Construction Engineering Co., Ltd., Wuhan 430014, China

**Keywords:** foundation pit, bracing, hybrid prefabricated reinforced concrete–steel structure foundation pit bracing system, numerical simulation

## Abstract

To overcome the inherent drawbacks of cast-in-place reinforced concrete bracing—such as long construction periods and difficult demolition—as well as the relatively high construction cost of steel structure bracing, while fully incorporating the respective technical advantages of these two traditional support systems, this paper proposes a novel hybrid prefabricated reinforced concrete (RC)–steel structure foundation pit bracing system. In order to investigate the overall bearing capacity variation in the standard components of this structure under complex external forces in foundation pits, a numerical model was established using the finite element software ABAQUS. The study examines the trend of the axial compressive bearing capacity of a single standard beam segment as the steel thickness of its external stiffening sleeve varies, as well as the effects of eccentric loading, oblique loading, and the presence or absence of auxiliary supports on the structural bearing capacity of multi-segment beam assemblies. The numerical analysis results show that the bearing capacity of a single beam segment exhibits a strong correlation with the variation in sleeve thickness, and a fitting curve of compressive strength as a function of thickness was derived. For the multi-segment assembly, an increase of 1 mm in the load eccentricity in the Y and Z directions reduces the ultimate peak load by approximately 20.95 kN and 23.94 kN, respectively; in the XY and XZ planes, an increase of 1° in the eccentric angle of the oblique load reduces the peak ultimate bearing capacity by about 6.02 kN and 9.67 kN, respectively. Auxiliary supports have a relatively minor influence on the structural bearing capacity. This research thoroughly explores the bearing capacity of the prefabricated steel–concrete composite and steel structure foundation pit bracing under complex working loads, providing strong support for engineering design and demonstrating broad application prospects.

## 1. Introduction

The ongoing acceleration of urban underground space development, combined with the large-scale implementation of major linear projects such as high-speed railways, urban rail transit, and utility tunnels, has made deep excavation engineering an indispensable core component of underground construction. This progress creates vast opportunities for innovation in excavation technology, but it also imposes increasingly severe engineering challenges [1]. Today’s urban deep excavations are typically characterized by large excavation scales, great depths, tight schedules, and sensitive, densely built surroundings. As a result, construction risk sources have multiplied considerably. Accidents such as excavation instability and excessive deformation occur frequently. These incidents not only pose major safety hazards to on-site construction but also present new challenges to theoretical research and technological optimization [2,3,4].

Excavation disturbs the original stress equilibrium in the surrounding soil, which induces deformation of the diaphragm wall. This deformation continues to grow as the excavation depth increases [5,6]. The design of an excavation support system must integrate multiple factors, including retaining structure characteristics, site geology, and excavation scale. Both qualitative and quantitative analyses are required to determine a suitable internal bracing configuration [7]. Internal bracing serves as the core strategy for controlling deformation in deep excavations. Support systems that combine diaphragm walls with internal bracing or soldier piles have been widely adopted. These internal braces generally fall into two categories: steel braces and concrete braces [8,9,10,11]. Prefabricated steel braces are easy to assemble and dismantle, offering notable schedule advantages, yet they exhibit inherent weaknesses in overall stability and joint performance [12,13]. Cast-in-place concrete braces provide excellent stiffness and deformation control, but they come with intrinsic drawbacks such as complex procedures, long construction periods, difficult demolition, and poor environmental compatibility [14,15].

As discussed above, the end joints of steel braces are inherently weak and susceptible to local damage, which can trigger progressive collapse of the excavation. In contrast, concrete braces with cast-in-place end joints exhibit strong connectivity, capable of resisting both compressive and tensile forces, making them more durable and reliable. To overcome the limitations of both systems, many researchers have focused on developing novel structural braces, with concrete-filled steel tubes (CFSTs) emerging as a growing trend in excavation support. Guo [16] proposed a prefabricated multi-limb CFST internal bracing system. Its components are factory-prefabricated with adjustable lengths, allowing the system to accommodate various excavation widths. Guo [17] introduced a precast concrete-filled steel tube (PCFT) internal brace, an innovative design that exploits the favorable compressive performance of rectangular CFST sections. However, the core issue with the aforementioned CFST and PCFT systems is that the connection nodes between segments are primarily joined by on-site welding or flanged bolting, This connection design relies primarily on the strength reserves of the joints themselves and lacks an effective mechanism to accommodate uneven deformation and eccentric forces between segments. As a result, the connection points become the weak links in the entire support system, making them highly susceptible to stress concentration and premature joint failure. This not only inhibits the full realization of the components’ overall load-bearing capacity but also limits the widespread adoption of such prefabricated support systems in large-span, high-load excavation pits.

To overcome the limitations of existing technologies, this paper proposes a hybrid prefabricated RC–steel structure foundation pit bracing system [18]. The system features low cost, high recyclability, low carbon emissions, and ease of demolition. Its load-bearing mechanism is primarily formed by precast standard concrete beam segments and auxiliary steel bracing. A hydraulic jack adjustment system enables fine dimensional tuning and transfers forces from the capping beam. Based on this excavation support system, selected components are numerically simulated using ABAQUS. The study addresses three aspects. First, it investigates how the parameters of the standard beam segment and the thickness of the outer steel sleeve plate influence the axial compressive strength. Second, it examines the load–displacement curves and peak load trends of multi-segment composite assemblies under eccentric and inclined loads. Third, it evaluates the effect of auxiliary bracing on the load-bearing capacity of these multi-segment composite structures.

## 2. Prefabricated Steel–Concrete Bracing Structure for Excavations

**Figure 1:**
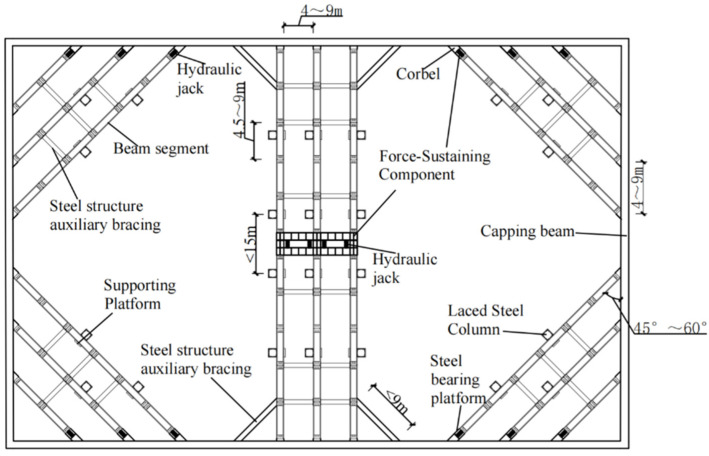
Plan view of a prefabricated RC–steel composite excavation bracing system (adapted from [18]).

The typical plan layout of the hybrid precast RC–steel (SS) excavation bracing system consists of capping beams, waling beams, reinforced concrete beam segments (hereinafter referred to as beam segments), auxiliary SS braces, corbels, steel tie columns, hydraulic jacks, steel bearing platforms, bracing platforms, and load-bearing components. In this system, the main brace—assembled from multiple beam segments—serves as the core load-carrying unit, while the individual beam segments act as the primary load-resisting elements. A schematic is provided in Figure 1.

**Figure 2:**
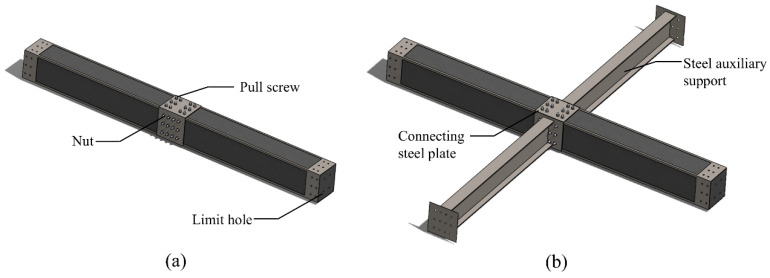
Schematic of beam segment connection details: (**a**) without auxiliary bracing, (**b**) with auxiliary bracing.

To ensure the overall stiffness and structural continuity of the main brace, reserved holes are provided at both ends of each beam segment. These holes enable reliable segment-to-segment splicing using steel plates and bolts, as well as coordinated connection with the auxiliary steel bracing. To strengthen the end-zone detailing and prevent concrete edge spalling during component reuse, a protective steel sleeve is installed around the concrete at the beam segment ends (the detailed configuration is presented later). This sleeve also provides local strengthening. From a load-resisting standpoint, the main brace (including its individual beam segments) primarily carries axial compression transferred from the excavation support system, accompanied by only minor shear forces and bending moments induced by self-weight. The auxiliary steel bracing bears a negligible share of the load; its core function is to enhance the overall stability of the bracing system. Figure 2 shows the connections between beam segments and an example with auxiliary steel bracing. The detailed configurations of the joints are described in the following sections.

As shown in Figure 1, the jack adjustment system used in this bracing system comprises a mid-span adjustment system and a beam end adjustment system. The mid-span adjustment system is designed for opposing braces that have splayed braces at their ends, and is installed at the mid-span of the main beam within such braces. The beam end adjustment system is intended for main braces without splayed braces at their ends, and is placed at the ends of these main braces. This adjustment system enables real-time adjustment of the entire structure, making it particularly suitable for complex excavation environments.

The bracing system adopts standardized beam segments paired with auxiliary steel bracing as internal support members, replacing cast-in-place reinforced concrete braces. This eliminates on-site rebar tying, concrete casting, and curing, which shortens the construction period. A hydraulic jack adjustment system and stay cable adjusters are provided, enabling adjustable and reliable support. Measures such as cast-in-place corbels and non-standard beam segments allow the system to accommodate excavations of different dimensions. The system uses standardized beam segments connected by through bolts, which simplifies installation and dismantling. As a result, the components can be reused multiple times, substantially reducing the carbon emissions of excavation support compared with cast-in-place reinforced concrete bracing. Consequently, the prefabricated RC–steel composite excavation bracing technology offers distinct advantages over both conventional cast-in-place reinforced concrete bracing and steel bracing. The system is well suited to medium-to-large excavations across a wide range of complex conditions.

## 3. Finite Element Model Development

### 3.1. Model Parameters

**Figure 3:**
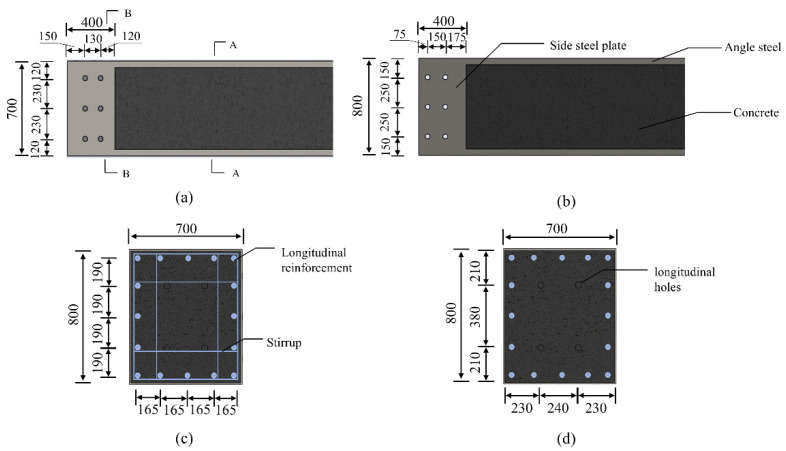
Schematic diagram of a beam segment (unit: mm): (**a**) vertical view of the beam segment, (**b**) side view of the beam segment, (**c**) section A-A, (**d**) section B-B.

This paper uses the ABAQUS 2021 finite element analysis software to establish a series of finite element models for prefabricated reinforced concrete beam segment used in lattice-type excavation shoring. The prefabricated beam sections have cross-sectional dimensions of 700 mm × 800 mm and a span of 4.5 m; To facilitate connection, bolt holes with a diameter of 40 mm are provided at the ends of the precast segments in both the transverse and longitudinal directions; the concrete strength grade is C35; the longitudinal reinforcement consists of HRB400 steel bars with a diameter of 22 mm; and the stirrups consist of HRB400 steel bars with a diameter of 8 mm. Due to the through holes at the ends of the beam segments, steel beam sleeves are used for reinforcement to offset the reduction in load-bearing capacity caused by the holes. These sleeves are fabricated by welding 10 mm-thick Q345 steel plates and ∠50 mm × 10 mm angle steel. The specific structural configuration and reinforcement layout of the beam segments are shown in Figure 3.

Steel plates are used to connect multiple beam sections. Before joining the two beam sections, stop bars are first inserted into the stop holes, followed by the installation of the bottom connecting plate. After aligning the beam sections, the top connecting plate is installed. The base material of the steel plates is 10 mm-thick Q345B low-alloy high-strength structural steel. The upper and lower plates are secured using through bolts made from 20mm-diameter HPB300 smooth surface rebar and M20 nuts. The lateral plates are connected in the same manner using the same materials; the only difference is that H-beams must be welded to the lateral plates where auxiliary supports are present. After the lateral plates are installed, they are welded to the upper and lower plates to form a single, integrated structure.

### 3.2. Cell Selection and Mesh Generation

**Figure 4:**
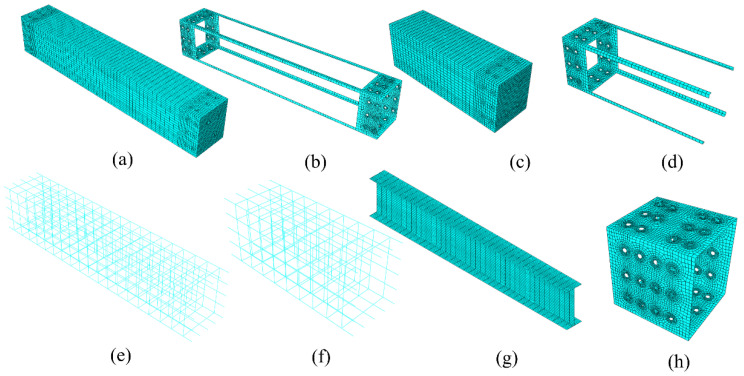
Schematic diagrams of the mesh divisions for each component. (**a**) Concrete beam, (**b**) beam sleeve, (**c**) 1/2 concrete beam, (**d**) 1/2 beam sleeve, (**e**) reinforcing steel cage, (**f**) 1/2 reinforcing steel cage, (**g**) H-beam brace, (**h**) connecting steel plate.

**Figure 5:**
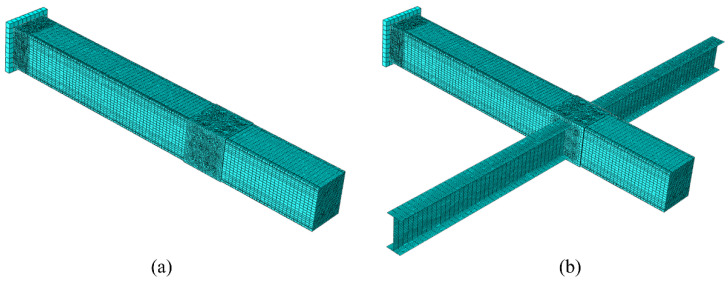
Overall mesh division for multi-beam sections. (**a**) Without auxiliary supports, (**b**) with auxiliary supports.

As described above, the finite element model will include concrete beams, reinforcing bars, beam sleeves, connecting plates, through bolts and nuts, H-beam braces, and loading plates. The concrete beams will be modeled using 3D solid elements (C3D8R). Since the thicknesses of the beam sleeves, connecting plates, and H-beam braces differ significantly from those of the solid structure, they will be modeled using S4R surface shell elements; the reinforcing bars within the beams will be modeled using T3D2 3D truss elements; to simplify the model, the function of the tie rods and nuts is to apply prestress, so the tie rods will be modeled using B31 two-node beam elements, and no nut components will be created. Since mesh size directly affects the convergence and computational efficiency of the analysis, and given the complex geometry of this structure, it is difficult to uniformly specify a single mesh size during the meshing process. Instead, the structure is divided into sections, and regional meshes are progressively created to achieve an optimal mesh configuration. An analysis of the mesh sizes for each component yielded the mesh size information shown in Table 1. After repeated testing, this meshing method ensures the reliability and accuracy of the results, reduces the likelihood of non-convergence, and improves computational efficiency. Figure 4 illustrates the mesh divisions for each component. In this study, when analyzing the splicing of multi-segment beams, a computational model is established using symmetry. Figure 4c–e show a standard beam end with a 1/2-length model, while Figure 5 depicts the mesh division of the model after splicing the multi-segment beams. The mesh size adopted in this study was selected after balancing structural applicability and computational efficiency. A mesh sensitivity analysis was not conducted. This is primarily because the structure is relatively complex and the meshing process is intricate, making it impractical to enforce a uniform prescribed mesh size throughout. Moreover, existing numerical studies on steel–concrete composite structures have not treated mesh sensitivity as a primary concern [19,20]. Therefore, the mesh used in this paper is considered adequately applicable to the analyzed component.

### 3.3. Contact Relationships

The interactions between the various components are simulated by defining appropriate contact relationships. In the finite element model presented in this paper, six types of contact relationships are defined. To reduce the complexity of the model, bond–slip between the concrete and reinforcing bars is accounted for by embedding the reinforcing bars within the concrete using the Embedded command. Since the concrete beam and the beam sleeve are cast as a single unit, a binding constraint is used to connect the two. The contact between the two concrete beam segments, the beam sleeve, and the connecting steel plate is defined as face-to-face contact. The tangential friction coefficient for the face-to-face contact pair on the concrete surface is 0.6 [21], while that for the steel plate surface is 0.3 [22]; the normal direction is set to “hard” contact for both. An MPC constraint is applied between the tension bolts and the connecting steel plate to apply the bolt preload. The H-beam auxiliary brace is directly connected to the connecting steel plate to establish an initial constraint; the loading plate and the concrete beam are connected using a binding constraint.

### 3.4. Boundary Conditions and Loading Methods

**Figure 6:**
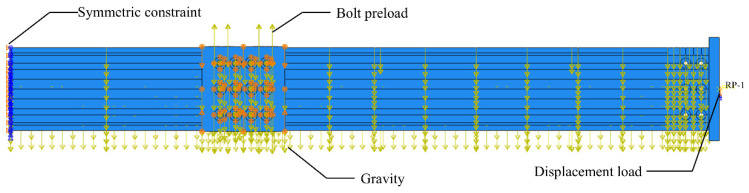
Constraints and loads.

To verify the agreement between the finite element analysis results and the experimental results, it is essential to ensure that the loading boundary conditions of the structure are consistent in both environments. Therefore, in this study, the boundary conditions and loading methods of the model were set based on the stress conditions of the beam sections in actual engineering applications. Due to symmetry, symmetric constraints were applied to the end faces of half of the concrete beam sections; for structures with bracing, fixed-end constraints were applied at the ends of the bracing. To prevent the model from failing to converge due to excessive loads, the loading method in this model employs a step-by-step, gradual approach. First, bolt pre-tensioning forces are applied at the beam segment connections, along with the effect of gravity; thus, the bolt pre-tensioning forces and gravity are applied first. A reference point (RP-1) is set at the center of the loading plate, and movement and rotation of the coupling point outside the horizontal plane are restricted. A horizontal displacement load is applied. The constraints and load application method of the numerical model are shown in Figure 6.

### 3.5. Constitutive Models

#### 3.5.1. Concrete Constitutive Model

For the concrete material model, this chapter adopts the concrete damaged plasticity (CDP) model built into the finite element software ABAQUS. The CDP model is a macroscopic phenomenological constitutive model originally proposed by Lubliner et al. [23] and later refined by Lee and Fenves [24]. It accounts for both plastic deformation and stiffness degradation, using the tensile damage variable $d_{t}$ and the compressive damage variable $d_{c}$ to describe damage evolution under tension and compression, respectively. The model is well suited for simulating the constitutive behavior of concrete under monotonic loading in this study. It can capture damage, crack propagation, and similar behaviors while offering good convergence.

**Figure 7:**
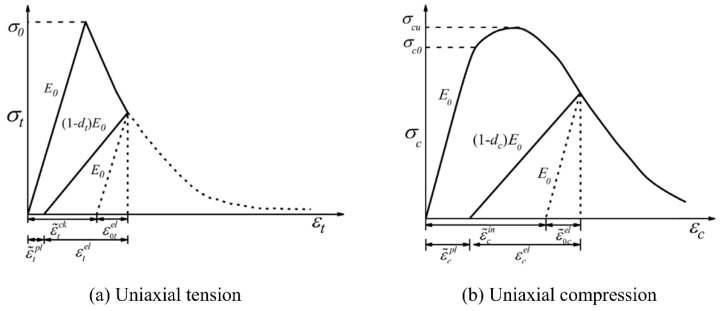
Stress–strain curve of concrete under uniaxial loading (adapted from ABAQUS [25]).

The CDP model primarily incorporates concrete damage variables into the uniaxial tensile and compressive stress–strain relationships. Figure 7 illustrates the mechanical behavior of concrete under uniaxial tension and compression, respectively. As a material characterized by high compressive strength and low tensile strength, concrete undergoes an elastic stage followed by a softening stage under tension, while under compression it experiences an elastic stage, a hardening stage, and a softening stage.

When inputting inelastic strain data into the CDP model in the software, a conversion based on the uniaxial stress–strain relationship of concrete is required. This stress–strain relationship adopts the uniaxial constitutive model for concrete given in the Chinese Code for Design of Concrete Structures (GB 50010-2010) [26] and can be expressed as:

(1) $$\left\{\begin{array}{c} \sigma_{t}=\left(1-d_{t}\right)E_{0}\varepsilon \;\\ \sigma_{c}=\left(1-d_{c}\right)E_{0}\varepsilon \end{array}\right.$$

where $\sigma_{t}$ and $\sigma_{c}$ denote the tensile and compressive stresses of concrete, and $d_{t}$ and $d_{c}$ are the tensile and compressive damage evolution parameters, with their respective calculation formulas given below.

(2) $$d_{t}=\left\{\begin{array}{cc} 1-\rho_{t}\left[1.2-0.2x^{5}\right] & x\leq 1\\ 1-\frac{\rho_{t}}{\alpha_{t}{\left(x-1\right)}^{1.7}+x} & x>1 \end{array}\right.$$

(3) $$d_{c}=\left\{\begin{array}{cc} 1-\frac{\rho_{c}n}{n-1+x^{n}} & x\leq 1\\ 1-\frac{\rho_{c}}{\alpha_{c}{\left(x-1\right)}^{2}+x} & x>1 \end{array}\right.$$

Here, $\alpha_{t}$ and $\alpha_{c}$ are the parameters governing the descending branches of the stress–strain curves. $\rho_{t}=\frac{f_{t,r}}{E_{c}\varepsilon_{t,r}}$ and $\rho_{c}=\frac{f_{c,r}}{E_{c}\varepsilon_{c,r}}$. For uniaxial tension, $x=\frac{\varepsilon }{\varepsilon_{t,r}}$; for uniaxial compression, $x=\frac{\varepsilon }{\varepsilon_{c,r}}$. $f_{t,r}$ and $f_{c,r}$ are the representative values of the uniaxial tensile and compressive strengths of concrete, respectively. $\varepsilon_{t,r}$ and $\varepsilon_{c,r}$ are the corresponding peak tensile and compressive strains. $\varepsilon_{c\left(t\right)}^{el}$ is the elastic strain of concrete in compression (or tension) accounting for damage, defined as $\varepsilon_{c\left(t\right)}^{el}=\frac{\sigma_{c\left(t\right)}}{\left(1-d_{c}\right)E_{0}}$. ${\tilde{\varepsilon }}_{c\left(t\right)}^{pl}$ denotes the plastic strain of concrete in compression (or tension) incorporating damage. ${\tilde{\varepsilon }}_{0c\left(t\right)}^{el}$ is the undamaged elastic strain of concrete in compression (or tension), given by ${\tilde{\varepsilon }}_{0c\left(t\right)}^{el}=\frac{\sigma_{c\left(t\right)}}{E_{0}}$. ${\tilde{\varepsilon }}_{c}^{in}$ and ${\tilde{\varepsilon }}_{t}^{ck}$ represent the inelastic strain in compression and the cracking strain in tension, respectively, n = 2.

The damage variable is a core parameter of the CDP model. The damage variable d, d is the general theoretical expression of the damage variable, the stress $\sigma_{c\left(t\right)}$, and the inelastic strain ${\tilde{\varepsilon }}_{0c\left(t\right)}^{el}$ are interrelated and together characterize the nonlinear behavior of concrete. In this study, the damage variable d is calculated using the formula derived from Sidoroff’s energy equivalence principle [27].

(4) $$d=1-\sqrt{\frac{\sigma_{c\left(t\right)}}{E_{0}\varepsilon_{c\left(t\right)}}}$$

When simulating concrete with the CDP model, the choice of other model parameters significantly affects the structural capacity and load–displacement curves. These parameters, as adopted in this study, are listed in Table 2. The mechanical properties of the concrete used in this study are shown in Table 3

When defining concrete constitutive parameters in ABAQUS, true stress and true strain must be used. The stress–strain relationship directly obtained from material tests, however, is in nominal (engineering) values. The conversion from nominal to true stress and strain is given as follows:

(5) $$\sigma =\sigma_{\mathrm{nom}}\left(1+\varepsilon_{\mathrm{nom}}\right)$$

(6) $$\varepsilon =\ln\left(1+\varepsilon_{\mathrm{nom}}\right)$$

where σ and ε denote the true stress and true strain, respectively; $\sigma_{\mathrm{nom}}$ and $\varepsilon_{\mathrm{nom}}$ denote the nominal stress and nominal strain, respectively.

**Figure 8:**
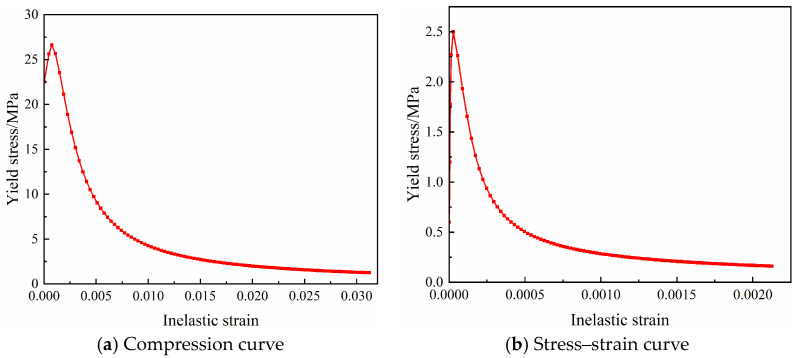
Stress–strain curve under compression and tension yielding—inelastic strain.

**Figure 9:**
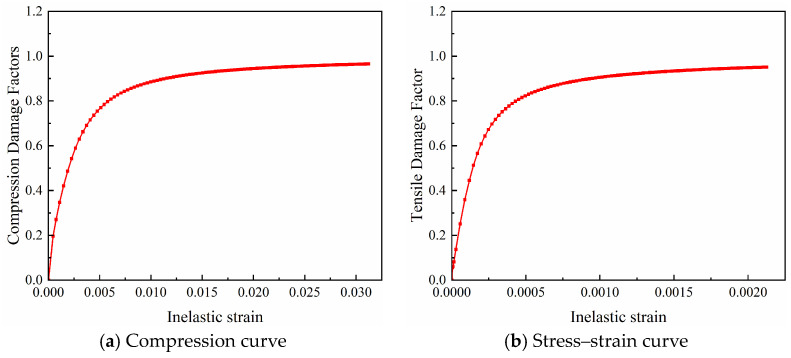
Compressive and tensile damage variable—inelastic strain curves.

Finally, since the C35 concrete used in the material tests of this study was commercial concrete with high consistency, the measured strength variation between different batches did not exceed 10%. By combining the relevant formulas presented in this section, the data shown in Figure 8 and Figure 9 were calculated and subsequently input into ABAQUS.

#### 3.5.2. Steel Constitutive Model

**Figure 10:**
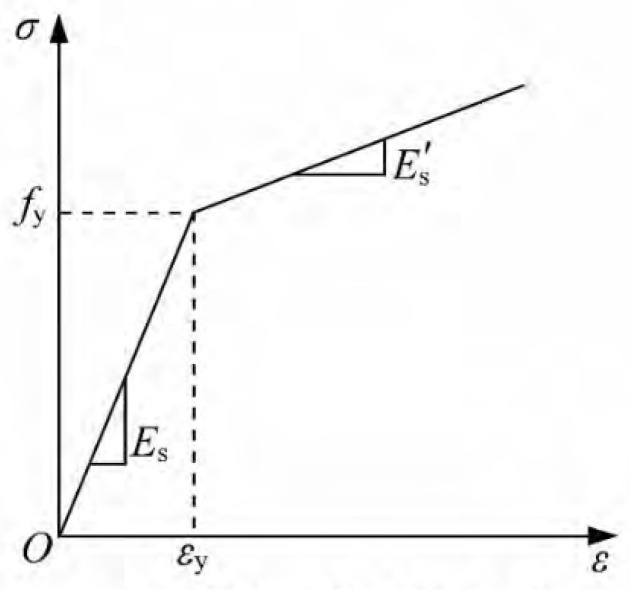
Constitutive relations of steel and reinforcing bars.

The stress–strain relationship of steel under monotonic tensile loading typically exhibits distinct elastic, yield plateau, and strain-hardening stages. To simplify the calculation and facilitate numerical implementation, the constitutive model for steel adopted in this study is a bilinear elastic–plastic hardening model, as shown in Figure 10. This model simplifies the stress–strain curve of steel into two linear segments: an elastic segment and a linear hardening segment, neglecting the minor effect of the yield plateau. It offers the advantages of requiring few parameters and good convergence and is widely used in nonlinear finite element analysis of reinforced concrete and steel structures. The steel parameters used in this study are listed in Table 4.

The uniaxial stress–strain relationship for steel is expressed using a piecewise function, as shown in Equation (7):

(7) $$\sigma_{s}=\left\{\begin{array}{cc} E_{s}\varepsilon_{s} & \left(\varepsilon_{s}\varepsilon_{y}\right)\\ f_{y}+k\left(\varepsilon_{s}-\varepsilon_{y}\right) & \left(\varepsilon_{y}<\varepsilon_{s}\leq \varepsilon_{u}\right) \end{array}\right.$$

where $E_{s}$ is the elastic modulus of steel; $\sigma_{s}$ is the steel stress; $f_{y}$ is the yield strength of steel; $\varepsilon_{s}$ is the steel strain; $\varepsilon_{y}$ is the yield strain of steel, $\varepsilon_{y}=f_{y}/E_{s}$; $\varepsilon_{u}$ is the ultimate strain of steel; and k is the slope of the hardening segment, defined as $k=\left(f_{u}-f_{y}\right)/\left(\varepsilon_{u}-\varepsilon_{y}\right)$, where $f_{u}$ is the ultimate tensile strength of steel.

### 3.6. Model Validation

The validity of a finite element analysis model is determined by comparing simulated values with experimental data; establishing an accurate finite element model is a critical step in studying numerical problems. The finite element software used in this paper is Abaqus 2021, and numerical calculations were performed using the Abaqus/Standard module included with the software. In this study, to further verify the accuracy of the finite element model, the axial compressive strength of an unperforated concrete beam obtained from numerical simulation was compared with the corresponding theoretical calculation values. Here, the concrete beam was meshed with a 50 mm grid, and the reinforcement was spaced at 100 mm. According to the provisions of the national standard “Code for Design of Concrete Structures” (GB 50010-2010) [26], the design value of the axial compressive strength of concrete, after accounting for the reduction factor, can be expressed as in Equation (7):

(8) $$N=0.9\varphi \left(f_{c}A+f_{y}^{\prime }A_{s}^{\prime }\right)$$

It should be noted that, in finite element analysis, the compressive strength parameter for concrete is typically taken as the characteristic value of axial compressive strength. Moreover, partial factors for load combinations and the member stability coefficient are not introduced in the calculation process. To ensure comparability between theoretical calculations and simulation results, the parameters in Equation (8) must be adjusted. Specifically, the design value of concrete compressive strength $f_{c}$ in Equation (8) is replaced by the characteristic value $f_{\mathrm{ck}}$, while the coefficient 0.9—which accounts for structural importance and load partial effects—and the member stability coefficient φ are omitted. Based on this modification, the axial compressive capacity of concrete without openings is calculated using Equation (9):

(9) $$N=f_{\mathrm{ck}}A+f_{y}^{\prime }A_{s}^{\prime }$$

In this equation, N is the compressive capacity of the specimen. A is the gross cross-sectional area, equal to 560,000 mm^2^. $f_{ck}$ is the characteristic axial compressive strength of concrete, taken as 24.3 N/mm^2^. $f_{y}^{\prime }$ is the design tensile strength of longitudinal reinforcement, taken as 360 N/mm^2^. $A_{s}^{\prime }$ is the total cross-sectional area of longitudinal reinforcement, equal to 6080 mm^2^.

**Figure 11:**
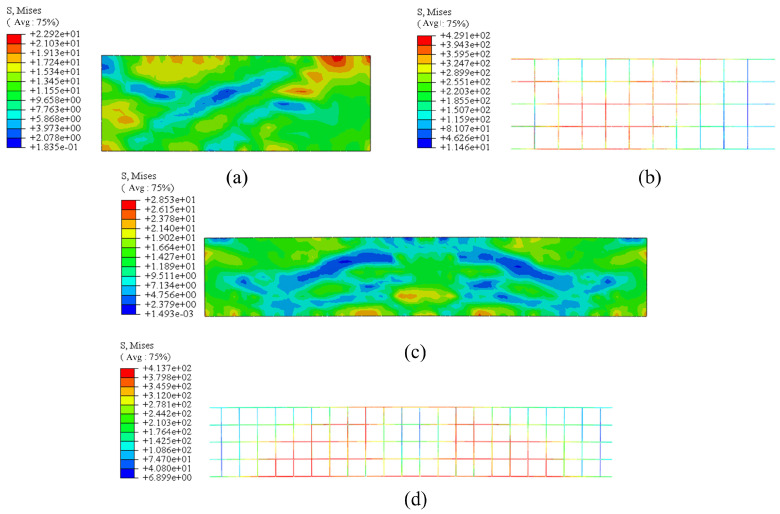
Stress contour plots for the symmetry verification beam. (**a**) Stress contour plot of the 1/2 beam, (**b**) stress contour plot of the 1/2 reinforcement cage, (**c**) stress contour plot of the beam, (**d**) stress contour plot of the reinforcement cage.

According to Equation (9), the theoretically calculated axial compressive capacity of the concrete beam specimen without perforations is 15,292.8 kN. Numerical simulation results indicate that the axial compressive strength of half of this structure is 14,824.3 kN, while the axial compressive strength of the beam structure without symmetry is 14,524.5 kN. The errors relative to the calculated value are 3.06% and 5.03%, respectively. The calculation results of the two models differ only slightly from the theoretical value and are both within an acceptable error range. The difference in numerical calculation values between the symmetric and asymmetric structures is 2.02%, which is negligible. As shown in the Mises stress contour plot comparing the two models in Figure 11, the stress distribution patterns, shear band locations, and damage zone ranges are completely consistent. Therefore, this demonstrates that the established finite element model is reliable, and the use of a half-structure for the numerical model does not compromise accuracy.

## 4. Discussion

### 4.1. The Effect of Beam Sleeve Thickness on the Load-Carrying Capacity of a Single Beam Segment

Beam sleeves enhance the axial compressive strength of precast beam segments. These sleeves comprise two components: end sleeves and intermediate angle irons. However, the strengthening mechanism of beam sleeves has yet to be systematically investigated. To address this, a finite element model with varying sleeve thicknesses was constructed, and axial compression tests were performed. Table 5 presents the axial compressive strengths for 17 different sleeve thicknesses. In this section, the effect of sleeve thickness on the numerical results is evaluated using the control variable method.

To facilitate the connection of precast components, holes were incorporated at the ends of the concrete beams. This design enables the connection of adjacent beam sections. However, excessive drilling reduces the axial compressive strength of the beams. Table 4, Item 1, shows that the beam with holes has a load-bearing capacity of 10,226.5 kN, representing a 31.02% reduction compared with a beam without holes. It can be seen that the holes have a significant impact on the overall strength of the concrete [29]. In this case, to improve the load-bearing capacity of the concrete beam sections, steel jacket plates are used, significantly enhancing both the overall strength and ductility [30]. Option 3 represents the beam sleeve combination used in the project. Compared to Option 1, the axial compressive strength increased by 10.44% and the overall ductility by 45.16%. This indicates that the use of beam sleeves improves the overall performance of concrete beams to some extent. However, to achieve the strength of a non-perforated beam, the No. 17 beam sleeve combination or thicker sleeves would be required. This would have a significant impact on the cost-effectiveness of the precast beam segments. Additionally, increased sleeve thickness would increase the overall weight of the beam segments, making the transportation of precast components relatively difficult.

**Figure 12:**
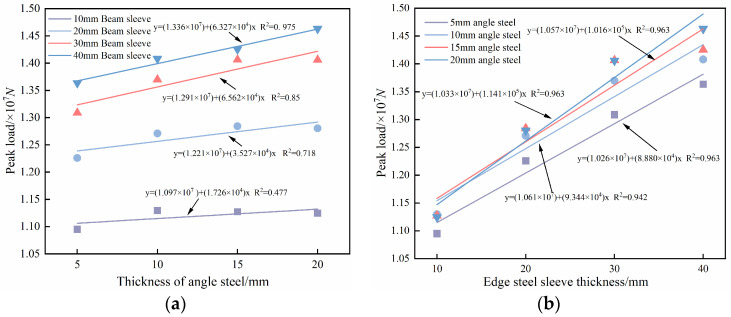
Graph showing the effect of beam sleeve thickness variations on the overall peak load. (**a**) Variation in the thickness of the end beam sleeve; (**b**) variations in the thickness of the center channel steel.

Figure 12 illustrates the trends in axial compressive strength as the thickness of the beam sleeve varies. Through two sets of single-factor controlled experiments, the influence of constraint member parameters on the ultimate peak axial compressive load of concrete beams was quantitatively investigated. Figure 12a and Figure 12b show the linear fitting results for the peak loads under different constraint parameters, respectively, as shown in Figure 12a. Overall, when the beam sleeve thickness is held constant, the peak load of the member exhibits a linear positive correlation with increasing angle steel thickness. The slopes of all fitted curves are positive, indicating that the reinforcement provided by the angle steel has a consistent positive effect on the axial ultimate load-carrying capacity of the concrete beam. However, the increase in strength for precast concrete beams is relatively limited; beyond 15 mm, the change in strength is not significant. Furthermore, for the same angle steel thickness, the peak load increases in a stepwise manner with increasing beam sleeve thickness; moreover, the greater the angle steel thickness, the more significant the difference in load-bearing capacity between different beam sleeve thicknesses, demonstrating the synergistic strengthening effect of the angle steel and beam sleeve confinement on the load-bearing capacity of the concrete beam. Figure 12b shows the variation in the peak load with the thickness of the end steel sleeve. Overall, when the angle steel thickness is fixed, the peak load of the member exhibits a highly significant linear positive correlation with increasing edge steel sleeve thickness. The coefficient of determination (R^2^ ≥ 0.942) for all test conditions indicates that the linear relationship between edge steel sleeve thickness and the axial ultimate load-carrying capacity of the concrete beam is extremely stable, and this trend is not affected by variations in the gradient of the angle steel thickness. Comparison of the two test sets demonstrates that both the end-sleeve thickness and the angle steel thickness positively affect the axial ultimate compressive capacity of the concrete beam, with a significant synergistic effect between them. Among these factors, the end-sleeve thickness enhances the load-bearing capacity significantly more than the angle steel thickness and exhibits a more stable linear relationship with capacity, thus serving as the primary parameter controlling the axial ultimate capacity of this concrete member. Increasing the end-sleeve thickness also markedly amplifies the strengthening effect provided by the angle steel confinement, a prerequisite for achieving efficient confinement and fully exploiting the reinforcing effect of the angle steel. From a physical mechanism perspective, the aforementioned pattern stems primarily from two factors: increasing the thickness of the angle steel enhances the lateral confinement stiffness of the core concrete, bringing it closer to a triaxial compression state and thereby linearly increasing the load-bearing capacity; however, when the angle steel thickness exceeds 15 mm, the corner confinement tends to reach saturation, and the gain diminishes. The beam sleeve and end steel sleeves provide continuous lateral support to the angle steel, suppressing local buckling during the later stages of loading and allowing the angle steel’s confinement capacity to be fully utilized; therefore, the greater the thickness of the steel sleeves, the more pronounced the synergistic strengthening effect.

**Figure 13:**
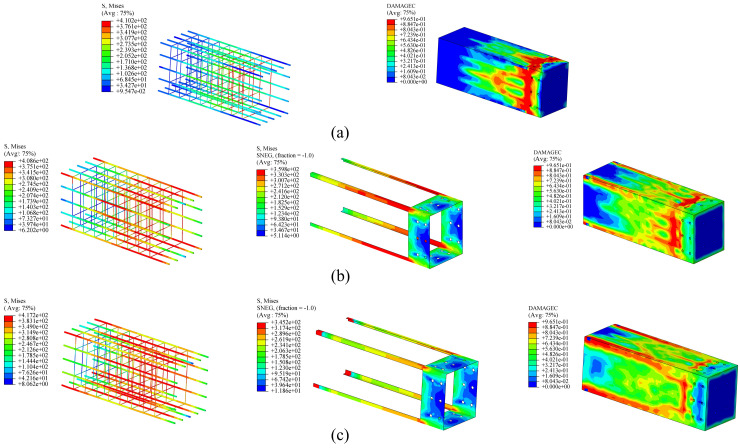
Contour plots of stress and damage distribution in concrete beams subjected to axial compression under different constraints from an outer beam sleeve: (**a**) 0 mm, (**b**) 10 mm, (**c**) 20 mm.

As shown in Figure 13, stress and damage contour plots were compared for two sets of concrete beam sections: one with a beam sleeve and one without. The stress distributions in the reinforcement cage and the beam sleeve were compared with the compressive damage distribution of the concrete to reveal the regulatory effects of the beam sleeve on the load-bearing mechanism, damage evolution, and failure mode. The stress contours of the reinforcement cage show that, without a beam sleeve, the overall stress level is low, with stress concentrations in the stirrups and low stresses in the longitudinal bars. Consequently, the strength potential of the reinforcement is not fully exploited, and the synergistic mechanism between the reinforcement and concrete is entirely lost. In the beam sleeve case, the stress distribution is uniform throughout the section, indicating that increased sleeve thickness enhances the confinement effect, amplifies the triaxial compression state in the core concrete, maximizes the strength potential of the reinforcement, and achieves a synergistic load-bearing mechanism among the reinforcement, concrete, and steel sleeve. A comparison of the concrete damage factors (DAMAGEC) across the three models shows that, in the beam without a sleeve, damage is concentrated at the beam ends and around the voids. This indicates that the voids significantly influence the failure pattern. In contrast, the beam sleeve confinement markedly suppresses damage development, substantially reducing the proportion of high-damage zones. Localized high-damage zones appear only in the mid-section of the beam. This indicates that the beam sleeve provides effective circumferential confinement, thereby delaying the propagation of microcracks. The resulting triaxial compression significantly enhances the compressive strength and deformation capacity of the core concrete, shifting the failure mode from unconfined brittle failure to confined ductile failure.

### 4.2. The Effect of Eccentric Loads on the Load-Carrying Capacity of Multiple Beam Sections

In actual engineering practice, when multiple precast beam segments are combined to form a lattice-type internal bracing system, installation errors often cause the system to bear eccentric loads [31]. To investigate the load-bearing capacity of multiple beam segments under eccentric loading, this paper designs nine sets of variables. The eccentricity variables in each axis direction are shown in Table 6. A numerical study is conducted on multiple precast concrete beam segments to determine the model’s eccentric compressive strength. In this model, the axial direction is the X-axis, so the eccentricity occurs in the Y- and Z-axis directions. With the center of the model as the coordinate origin, the eccentricity distances are all in the negative direction of each axis. Although gravitational loads act in the Y-axis direction, preliminary calculations indicate that their effect on the eccentricity direction is negligible; therefore, the analysis is conducted for a single direction along each axis.

**Figure 14:**
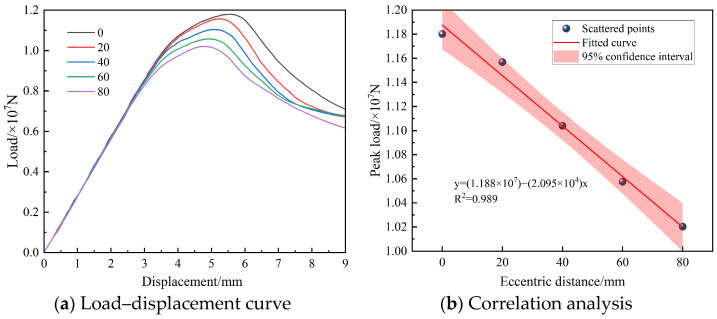
Correlation analysis chart of the Y-axis eccentric load displacement curves and peak loads.

**Figure 15:**
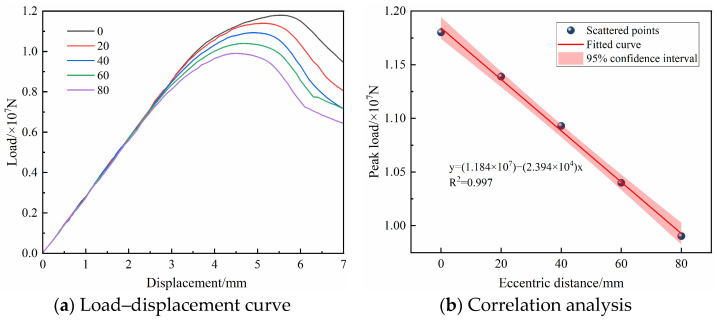
Correlation analysis chart of Z-axis eccentric load displacement curves and peak loads.

Table 6 shows that Specimen No. 1, tested under axial compression with multiple beam segments, exhibits higher strength than the single-segment specimen. This increase primarily results from the steel plate and bolt connections between segments, which strengthen the regions around the bolt holes and thus raise the overall strength. Figure 14 and Figure 15 present the load–displacement curves and the variation in peak strength with eccentricity for the multi-segment model under eccentric loading along the Y- and Z-axes, respectively. Linear trend lines are used to characterize the rate of strength degradation. At a displacement of 3 mm, the ascending branches of the load–displacement curves are nearly identical across different eccentricities. This occurs because both the steel sleeve and the concrete remain in the elastic stage, and the initial mechanical response is highly consistent regardless of eccentricity. This consistency also verifies the uniformity and stability of force transfer in the precast spliced segments during the elastic stage. As the load further increases, a larger eccentricity causes the peak load to appear earlier and become smaller. Both the ultimate bearing capacity and the ductility of the member deteriorate significantly with increasing eccentricity. The fitted curves reveal a very strong negative linear correlation between the peak load and the eccentricity along both the Y- and Z-axes. The resulting fitting formulas offer high prediction accuracy, with all R^2^ values exceeding 0.98. For every 1 mm increase in eccentricity, the ultimate axial peak load decreases by approximately 20.95 kN along the Y-axis and by approximately 23.94 kN along the Z-axis. This indicates that Z-axis eccentricity exerts a greater influence on the strength of the structure.

**Figure 16:**
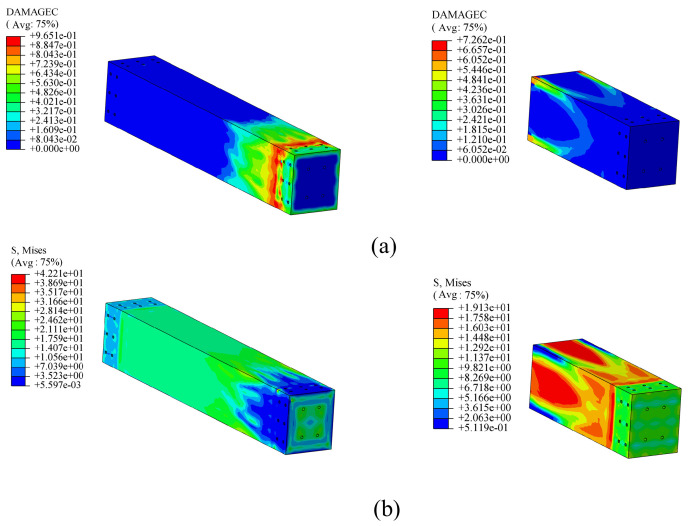
Contour plots of damage stresses in multi-beam sections under axial loading. (**a**) Contour plot of the damage factor distribution in a concrete beam under compression; (**b**) contour plot of the stress distribution in a concrete beam.

**Figure 17:**
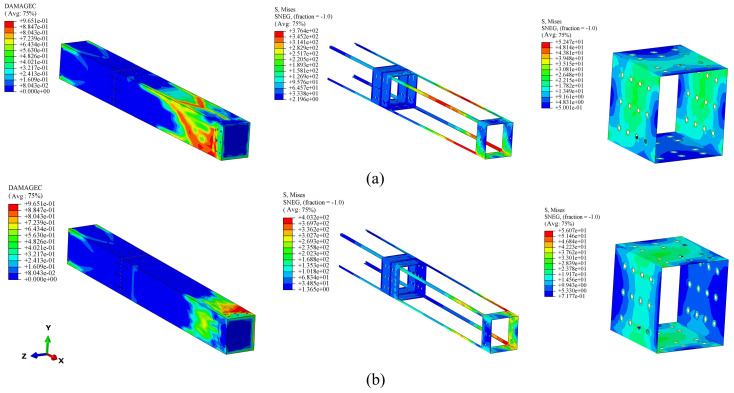
Contour plot distribution for multiple beam sections at an eccentricity of 40 mm. (**a**) negative Y-direction eccentricity; (**b**) negative Z-direction eccentricity.

Figure 16 shows the damage distribution contour plot and stress distribution contour plot for a concrete beam composed of multiple precast beam segments under axial loading. As shown in Figure 16a, the high-damage zone (DAMAGEC > 0.8) on the left beam segment is entirely concentrated at the loading end section, with a damage peak of 0.9651, approaching the limit state of complete material damage. This beam segment is also closely adjacent to the loading end, which is similar to the failure of a single beam segment’s axial compression at the loading end. The right-hand beam segment exhibits less damage. Since forces are transmitted through the connection plates or between the concrete beams, the load is also absorbed by the connection plates, resulting in relatively smaller forces being transmitted to the next beam segment. This is confirmed by Figure 16b, which shows that the stress distribution in the concrete at the loading end is significantly greater than that in the non-loading end beam segment. Overall, for structures composed of multiple beam segments, the load-bearing capacity of the loading end segment is critical; failure must first occur in this region. Figure 17 shows the stress–strain diagrams under ultimate loads for a variable model with a 40 mm eccentricity. All diagrams demonstrate lateral crushing failure of the concrete beam segments under eccentric loading. The concrete has already failed, and the concrete beam sections have long since yielded, while the connecting steel plates remain far from their yield strength. In actual engineering applications, reducing the thickness or strength of the connecting steel plates would have a minimal impact on the overall structure.

### 4.3. The Effect of Oblique Loads on the Load-Carrying Capacity of Multiple Beam Sections

In actual engineering practice, the installation of precast internal bracing in excavation shoring systems is never subject to ideal, purely axial compressive loads. The components are inevitably influenced by external factors, causing the final load configuration to transform into an oblique load at a certain angle to the component’s axis [32]. To investigate changes in the mechanical properties of these members under oblique loading, this study established 10 sets of oblique load variables. These were divided into five sets for the XY plane (load components in the X and Y directions) and five sets for the XZ plane (load components in the X and Z directions), each ranging from 10° to 50°. The oblique compressive strength was calculated through numerical simulation, as shown in Table 7.

**Figure 18:**
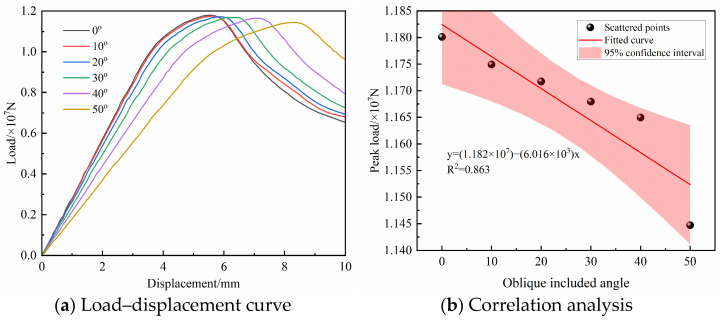
Load–displacement curves and linear analysis diagrams under oblique loads in the XY plane.

**Figure 19:**
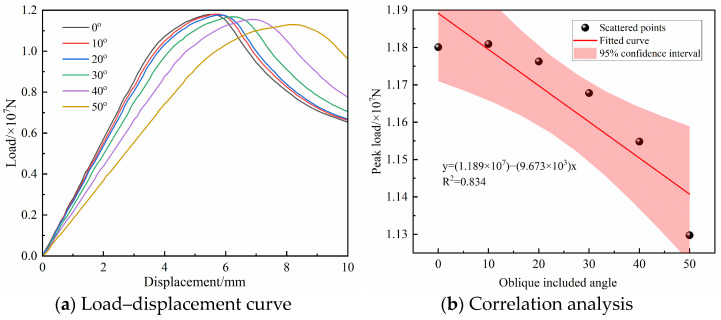
Load–displacement curves and linear analysis diagrams under oblique loads in the XZ plane.

**Figure 20:**
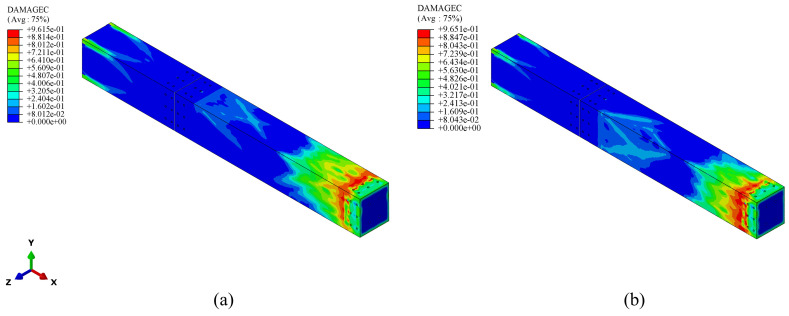
Compressive failure diagrams of concrete beam segments under a 50° oblique load: (**a**) XY plane tilt, (**b**) XZ plane tilt.

The results of these two sets of numerical simulations systematically reveal the influence of the eccentric angle of oblique loading on the load–displacement behavior, ultimate load-carrying capacity, and failure modes of multi-bolt-connected precast members in the XY and XZ orthogonal planes. They also quantify the relationship between the eccentric angle and the key mechanical properties of the members, as shown in Figure 16 and Figure 17. Figure 18a and Figure 19a display the load–displacement curves of the structure under varying oblique angles. As the eccentric angle increases, the peak load continues to decrease; under a 50° eccentric condition, the peak load-bearing capacity decreases by 3.5–4.1% compared to the 0° reference group. The slope of the elastic stage curve decreases slightly with increasing eccentricity angle, indicating that the bending deformation induced by the lateral eccentricity component causes the equivalent axial stiffness of the member to gradually decrease as the eccentricity angle increases. The ultimate displacement corresponding to the peak load increases with increasing eccentricity angle, and the curve in the yielding stage tends to flatten. This indicates that the eccentric loading effectively mitigates the brittle failure characteristics of the member, allowing the tensile resistance of the steel beam assembly to manifest, thereby enhancing the member’s deformation capacity and energy dissipation capability. Figure 18b and Figure 19b present the results of a univariate linear regression analysis, with the eccentric angle of the oblique load as the independent variable and the peak ultimate load-bearing capacity of the member as the dependent variable. This analysis quantifies the relationship between the eccentric angle and the ultimate load-bearing capacity for both planes, and the fitting results demonstrate good statistical significance and engineering applicability. In the XY and XZ planes, for every 1° increase in the eccentric angle of the oblique load, the peak ultimate load-carrying capacity of the member decreases by approximately 6.02 kN and 9.67 kN, respectively. The rate of decline in the XZ plane is 60.6% higher than that in the XY plane, indicating that members in the XZ plane are more sensitive to lateral eccentric loads, primarily due to the smaller cross-sectional width in that direction. It can also be observed that the rate of decline is greatest in both directions between 40° and 50°, indicating that when the oblique load angle exceeds 40°, it has a significant impact on the structural bearing capacity and should therefore be avoided. Figure 20 illustrates the compressive failure of concrete under oblique loading. As shown in the figure, the oblique load causes more pronounced compressive failure on one side of the concrete end face, primarily concentrated at the end where the load is applied, while the central section of the concrete beam exhibits lower levels of failure.

### 4.4. The Effect of Auxiliary Support Structures on the Load-Carrying Capacity of Multiple Beam Sections

In an engineering context, individual components do not typically exist in isolation within a system; rather, they work together to form the reinforced concrete–steel foundation pit shoring system discussed in this paper. In this system, steel structural components primarily serve as auxiliary supports. To investigate the impact of auxiliary supports on the overall load-bearing capacity of the structure, this section will compare changes in the load-bearing capacity of a composite structure consisting of multiple beam segments with and without auxiliary supports. Since auxiliary supports primarily affect the lateral deformation of the structure, this subsection employs an eccentric load to compare the load-bearing capacity of the structure under an 80 mm eccentricity condition.

**Figure 21:**
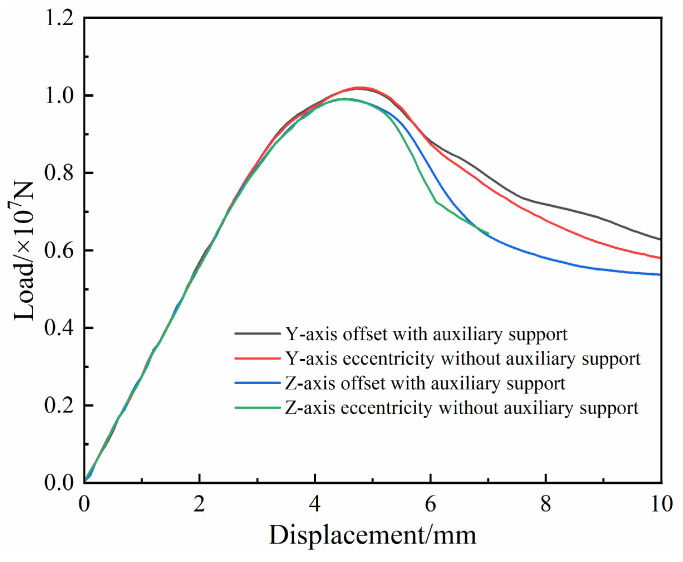
Load–displacement curves with and without auxiliary support members.

**Figure 22:**
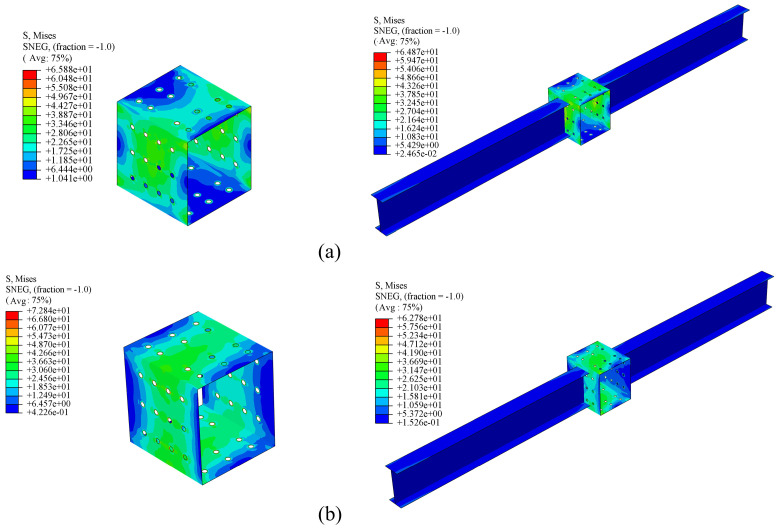
Stress contour plots of connection members with and without beam sleeves: (**a**) Y-axis eccentricity, (**b**) Z-axis eccentricity.

Figure 21 shows the load–displacement curves for multiple beam segments under eccentrically loaded compression, with and without auxiliary supports. From the curves, it can be seen that the effect of auxiliary supports on the elastic rise phase and peak load of multiple beam segments is almost negligible, and there is essentially no significant difference in peak load. This is primarily because the failure plane of the structure is mainly concentrated at the loading end; at this point, the force transmitted by the structure to the subsequent members gradually decreases, rendering the auxiliary supports ineffective in enhancing the overall load-bearing capacity. This is also confirmed by Figure 17 in Section 4.2. After the peak load–displacement point, it can be observed that the decline in eccentric compression strength with auxiliary supports is smaller than that without beam sleeves, indicating that auxiliary supports enhance the structure’s resistance to deformation. In design, auxiliary supports serve to ensure overall stability. Figure 22 displays the stress contour distribution of the connection members at the beam ends with and without the beam sleeve. The figure shows that the auxiliary support reduces the maximum stress in the connection members, with the greatest reduction occurring under Z-axis eccentric loading, amounting to 13.81%. Although the maximum stress in this structure does not reach the yield strength of the members, the material usage of the connection members can be optimized in subsequent design iterations. Regarding the auxiliary bracing in the steel structure, the stress distribution in the bracing is relatively low under eccentric loading, which confirms that the auxiliary bracing has a minimal impact on the structural load-bearing capacity under this operating condition.

## 5. Conclusions

To address the shortcomings of existing excavation shoring techniques, this paper proposes a prefabricated reinforced concrete–steel excavation shoring system. Additionally, to investigate the stress conditions of certain components in this structure during service, a computational model of the structure under complex loading conditions was developed using numerical simulation techniques. The results of this model are as follows:

(1) Due to connection requirements, standard beam sections have numerous holes at their ends, which reduce the axial load-bearing capacity of the beam sections by 31.02%. The addition of steel beam sleeves improves the structural load-bearing capacity. By varying the thickness of the steel plates at the ends of the sleeves and the thickness of the angle steel in the middle, it was found that the thickness of the end steel plates plays a critical role in the load-bearing capacity of standard beam sections.
(2) Joining multiple standard beam segments improves the structure’s axial load-bearing capacity. However, under eccentric loading, the load-bearing capacity of the assembled beams gradually decreases. For every 1 mm increase in the eccentricity of the Y- and Z-directional loads, the axial ultimate peak load of the prefabricated assembled beam decreases by approximately 20.95 kN and 23.94 kN, respectively.
(3) Under oblique loading, the combined structure of multiple beam segments will experience increased deformation at the peak load, and the peak load will gradually decrease. In the XY and XZ planes, for every 1° increase in the eccentric angle of the oblique load, the peak ultimate load-bearing capacity of the member decreases by approximately 6.02 kN and 9.67 kN, respectively.
(4) Based on the failure patterns observed under various loading conditions, structural failure is primarily concentrated at the loading end of the beam segments. To improve the structural load-carrying capacity, it is necessary to enhance the strength of the standard beam segments at the ends.

Although this paper has examined the behavior of multi-segment beam assemblies under complex loading conditions, the analysis of structures with auxiliary bracing is limited to the most unfavorable eccentric load case among the investigated factors. Due to space limitations, subsequent work will focus on the load-bearing capacity of structures with auxiliary bracing under various coupled complex loading conditions.

## Figures and Tables

**Table 1:** Component grid size (mm).

Part Name	Unit Type	Shortest Side	Longest Side	Average Side Length
Concrete beam	C3D8R	7.50	96.11	64.50
Reinforcing bars	T3D2	50.0	50.0	50.0
Beam sleeve	S4R	3.52	70.00	24.86
Connecting plate	S4R	3.14	15.92	49.02
Tension bolts	B31	100	100	100
H-beam brace	S4R	4.32	52.10	100.2

**Table 2:** Selection of additional parameters for the concrete damaged plasticity model [28].

Expansion Angle	Eccentricity	Ratio of Biaxial to Uniaxial Compressive Strength	Yield Surface Parameter	Viscosity Coefficient
30	0.1	1.16	0.667	0.001

**Table 3:** Properties of concrete materials.

Strength Grade	Standard Value of Axial Compressive Strength $f_{ck}/MPa$	Standard Value for Axial Tensile Strength $f_{tk}/MPa$	Modulus of Elasticity $E_{s}/MPa$	Poisson’s Ratio
C35	23.5	2.21	31,457	0.2

**Table 4:** Material properties of steel and reinforcing bars.

Component	Material Grade	Yield Strength *f_y_*/MPa	Ultimate Strength *f_u_*/MPa	Modulus of Elasticity *E_s_*/MPa
Longitudinal reinforcement	HRB400	400	540	2.00 × 10^5^
Stirrups	HRB400	400	540	2.00 × 10^5^
Beam sleeve	Q345B	345	560	2.06 × 10^5^
Connecting plate	Q345B	345	560	2.06 × 10^5^
H-beam brace	Q345B	345	560	2.06 × 10^5^
Tension bolts	HPB300	300	420	2.10 × 10^5^

**Table 5:** Thickness of steel plates in beam sleeves in different regions; axial compressive strength of precast beam segments.

Number	Thickness of the End Beam Sleeve/mm	Angle Steel Thickness/mm	Axial Compressive Strength /KN	Peak Displacement /mm
1	0	0	10,226.5	1.55
2	10	5	10,950.1	2.25
3		10	11,294.2	2.25
4		15	11,271.7	2.1
5		20	11,245.3	1.95
6	20	5	12,258.8	2.35
7		10	12,709.6	2.5
8		15	12,841.9	2.4
9		20	12,802.5	2.4
10	30	5	13,087.7	2.5
11		10	13,696.4	2.7
12		15	14,062.5	2.6
13		20	14,058.7	2.6
14	40	5	13,633.9	2.45
15		10	14,079.8	2.5
16		15	14,251.7	2.5
17		20	14,631.1	2.6

**Table 6:** Eccentric compressive strength of multiple precast beam segments under eccentric loading.

Number	Eccentric Direction	Eccentricity/mm	Eccentric Compressive Strength/KN
1	-	0	11,800.8
2	Negative direction of the Y-axis	20	11,568.0
3		40	11,039.2
4		60	10,574.8
5		80	10,202.8
6	Negative direction of the Z-axis	20	11,389.2
7		40	10,929.5
8		60	10,399.0
9		80	9902.3

**Table 7:** Diagonal compressive strength of multiple prefabricated beam segments under diagonal loads.

Number	Inclined Plane	Angle of Inclination	Diagonal Compressive Strength/KN
1	XY plane	10	11,749.1
2		20	11,717.3
3		30	11,679.5
4		40	11,649.5
5		50	11,447.0
6	XZ plane	10	11,809.2
7		20	11,762.6
8		30	11,677.9
9		40	11,548.1
10		50	11,297.3

## Data Availability

The original contributions presented in this study are included in the article. Further inquiries can be directed to the corresponding author.
